# Water Absorption Kinetics in Natural Rubber Composites Reinforced with Natural Fibers Processed by Electron Beam Irradiation

**DOI:** 10.3390/polym12112437

**Published:** 2020-10-22

**Authors:** Elena Manaila, Gabriela Craciun, Daniel Ighigeanu

**Affiliations:** National Institute for Laser, Plasma and Radiation Physics, 409 Atomistilor St., 077125 Magurele, Romania; elena.manaila@inflpr.ro (E.M.); gabriela.craciun@inflpr.ro (G.C.)

**Keywords:** natural rubber, natural fibers, composites, electron beam irradiation, water absorption kinetics

## Abstract

Natural rubber composites reinforced with hemp, flax, and wood sawdust were obtained by irradiation at room temperature with an electron beam of 5.5 MeV in order to meet the actual need for new materials that are environmentally friendly and safe for human health. The natural fibers loading was between 5 and 20 phr and the processing doses were between 75 and 600 kGy. The kinetics of water absorption in these materials were studied. The water diffusion was analyzed through Fick’s law. The water absorption parameters (Qt and Qeq), diffusion parameters (k and n), diffusion coefficient (D), intrinsic diffusion coefficient (D*), sorption coefficient (S), and permeation coefficient (P) have depended on the fiber nature, amount used in blends, and irradiation dose. The obtained results showed that not in the case of each type of fiber used, the water absorption could be correlated with the specific cellulose and hemicellulose content, due to the changes induced by the electron beam.

## 1. Introduction

Natural rubber (NR) is a green and renewable industrial raw material, commonly used for manufacturing a wide variety of products ranging from medical devices and personal protective equipment to aircraft tires. Vulcanization/cross-linking, the most important stage in NR processing for getting much more resistant and elastic products, is the chemical process performed by the addition of 2–3% sulfur in the presence of organic accelerators [1,2]. The process takes place under the influence of time, temperature, and pressure, and, depending on them and the chosen vulcanization system, can be obtained cross-links as mono-, di-, tri-, or higher poly-sulfides. Unfortunately, these compounds together with their reaction products are cytotoxic and responsible for many forms of allergies [3]. Currently, the curing system using peroxides is used as a viable alternative especially if there are targeted performances like purity, good resistance to high temperature, shelf life, good elastic and electrical properties, low compression set, and no discoloration of finished products [4,5,6].

The cross-linking and grafting of the elastomers by irradiation has been promoted and is now in use due to the advantages of the process in terms of cleanliness, homogeneity, no heat and vulcanizing agents addition (sulfur or peroxides). Especially by the use of electron beam (EB) irradiation at room temperature in NR curing, many other technical advantages can be noticed: short processing time, cross-linking degree controlled trough the irradiation dose, no oxidative degeneration in polymers, high productivity and low material waste [6,7].

In many industrial applications, NR with special properties is needed and for these, reinforcing fillers as carbon black or silica are used [8]. Even if the NR properties become remarkable, it cannot be overlooked that they have a harmful effect on human health that consists of silicosis, cancer, autoimmune diseases, tuberculosis, kidney diseases, etc. [9,10]. Human health together with the care for the environment, are solid reasons for the interest in the development of eco-composites such as natural fibers. So, qualities such as low cost, biodegradability, low density, high filling levels, rigidity, and mechanical resistance comparable to those of fiberglass, and availability in a wide variety all over the world, places the replacement of synthetic fibers with natural fibers at the top of the research topics [11,12].

Between animal, mineral, and vegetal fibers, the last category is the most commonly accepted by industries and, for this reason, the most analyzed. Cellulose seems to be the main constituent found responsible for eco-composites special mechanical properties [13]. This can be found in plant stems (flax, hemp, jute, kenaf, and ramie), leaves (abaca, banana, pineapple, and sisal), seeds (coir, cotton, and kapok), roots, and wood [14]. The most popular reinforcement materials used to obtain eco-composites are flax, hemp, and wood sawdust.

The properties of polymeric composites reinforced with natural fibers are strongly influenced by the degree of fiber adhesion to the polymeric matrix (due to the hydrophilic nature of the cellulosic fibers opposite to the hydrophobic nature of the polymer matrix), stretching and arrangement, thickness, length or amount [15,16,17]. For example, the moisture absorption leads to dimensional changes and micro-cracking, degradation of the fiber-matrix interface region and consequently, to reduction of the composites mechanical properties. The problem must be analyzed and solved so that natural fibers to be considered favorable for the reinforcement of materials for outdoor utilization [12,18,19,20]. The water absorption is mainly measured by polymer weight gain and its diffusion analyzed through Fick’s law, that pointed out three different mechanisms involved: the first one connected with the penetration of water molecules between polymer chains inside micro-gaps, the second one with the capillary transport into the gaps and flaws at the fiber-matrix interfaces and the third with the transport through micro-cracks in the matrix formed during the compounding process [19,21,22,23,24]. Also, the reactive functional groups of the fibers are covered by waxy and pectin substances which affect the interpenetration with the polymeric matrix. A solution to increase the effectiveness of interfacial bonding and thus, to improve the mechanical properties is the treatment of the fiber surface by different chemical methods, such as: the monomers; grafting, bleaching, acetylation, etc. [16,25]. These chemical treatments increase the roughness of the fiber surface and help to stop the process of moisture absorption. The compatibility between composite components can be also improved by using coupling agents that contain functional groups grafted onto the chain of the polymer. Other treatment techniques for a fiber’s surface include peroxide [16,26], plasma [16,27,28,29,30], or electron beam [31,32,33,34]. Organic peroxides are easily decomposed in free radicals, which further go on to react with the cellulose of the fiber and hydrogen group of the matrix [26]. The plasma treatment is very efficient to modify the surface of natural polymers without any change in their bulk properties [27,28,29,30]. Ionizing radiation treatment is generally short and no chemicals are needed [31].

The goal of the paper is to study the kinetics of water absorption, characteristic to hemp, flax, and wood sawdust used as reinforcement fillers for natural rubber vulcanized by electron beam irradiation.

## 2. Materials and Methods

### 2.1. Materials

The raw materials used in experiments were as follows: natural rubber of Crep 1X type (from Almar Trading Co (Pte) Ltd., Malwatte Valley Plantations PLC, Colombo, Sri Lanka), pentaerythritol tetrakis (3-(3,5-di-tert-butyl-4-hydroxyphenyl) propionate Irganox 1010 (from BASF Schweiz, Ciudad de Mexico, Mexico), polyethylene glycol PEG 4000 (from Advance Petrochemicals Ltd., Ahmedabad, Gujarat, India), hemp, flax, and wood sawdust used as fillers. The sawdust of beech wood was obtained from a local sawmill in Romania. Sawdust particle properties in terms of dimension (mesh 250–270) and purity (derived from a single type of wood), were maintained in all experiments. The hemp was bought from a profile store and was mechanically combed. The flax, in form of waste, was purchased from a textile factory. The flax and hemp fibers were cut to a length of maximum 3 mm. The main properties of materials used in experiments are presented in Table 1.

### 2.2. Sample Preparation

The composition of the blends used in study and the samples codes are shown in Table 2 and Table 3, respectively. Natural rubber blends were prepared on a laboratory electrically heated two-roll mill machine, equipped with cooling system. The working parameters were presented in previous works [41,42,43,44,45].

### 2.3. Experimental Installation and Samples Irradiation

The samples were prepared for irradiation by packaging in polyethylene film in order to minimize the oxidation and then irradiated at 75, 150, 300, and 600 kGy in atmospheric conditions and at room temperature of 25 °C, using the linear electron beam accelerator called ALID 7. Details about samples preparation, irradiation procedure and installation description are presented in previous studies [41,42,43,44,45].

### 2.4. Laboratory Tests—Water Absorption Tests and Measurements

Water uptake tests in accordance with ISO 20344/2011 were made in order to investigate the effect of water absorption on the properties of NR rubber composites reinforced with natural fiber. Water absorption experiments were done on circular samples of 30-mm diameter and 2-mm thickness. At least five tests were realized for every group/type of samples. Before initial weighing, all samples were dried for 24 h in a laboratory oven at 80 °C and then placed in desiccator for cooling. Water absorption tests were conducted by immersing the samples in distilled water in closed glass containers and keeping them at room temperature of 23 ± 2 °C. The samples were taken out from the glass containers at regular time intervals, the wet surfaces were quickly wiped using a tissue paper and weighed until no further increase in water absorption was detected. The weighing was done in bottle with stopper and the weighing process of every sample was no longer than 10 s. The weighing precision was within 0.1 mg.

## 3. Results and Discussion

### 3.1. Water Absorption Behavior

In order to obtain NR rubber eco-composites for industrial use, a number of disadvantages related to the use of fibers as filler must be overcome. The poor adhesion between the hydrophobic NR matrix and fibers or the difference between the water absorption mode of the rubber matrix compared to that of the fibers, constitute drawbacks to be solved before the widespread use of eco-composites [46,47,48,49]. The natural fibers are composed of cellulose, hemicellulose, lignin, pectin, wax, water-soluble substances, and fat. Cellulose, considered as the main constituent in the fiber structure, is the one that imparts its stiffness, strength, structural stability, and is especially responsible for moisture absorption. On the other hand, lignin is the element that acts as a natural effective shield against environmental circumstances such as humidity and temperature [46,50,51,52].

The water absorption in the composites at the time *t*, *Q_t_*, expressed in mol%, has been calculated by difference between the weights of samples immersed in water and dry samples, using the following Equation (1) [53]:

(1) $$Q_{t}=\frac{\left(W_{t}-W_{0}\right)/M_{W}}{W_{0}}\times 100$$

where *W_t_* and *W*_0_ are the weights of the samples immersed in water for time *t* and dry samples; *M_w_* is the solvent molar mass (water in this case, 18.0153 g/mol).

**Figure 1:**
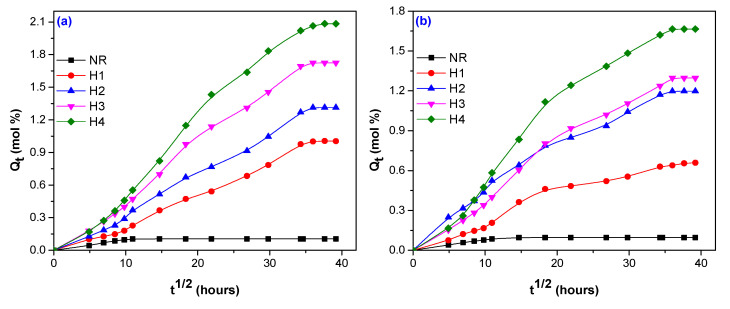

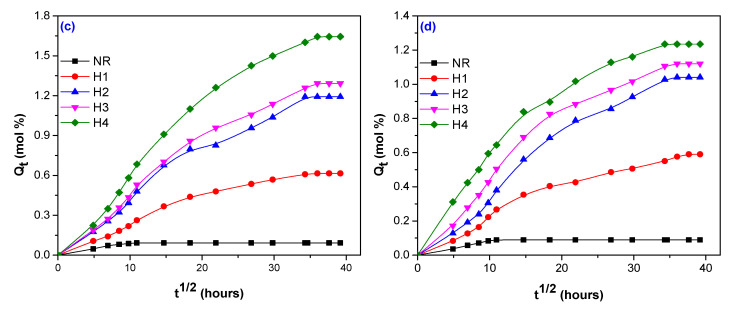
*Q_t_* variation versus immersion time for blends based on Natural rubber (NR) and hemp, irradiated at 75 kGy (**a**); 150 kGy (**b**); 300 kGy (**c**)**;** and 600 kGy (**d**).

**Figure 2:**
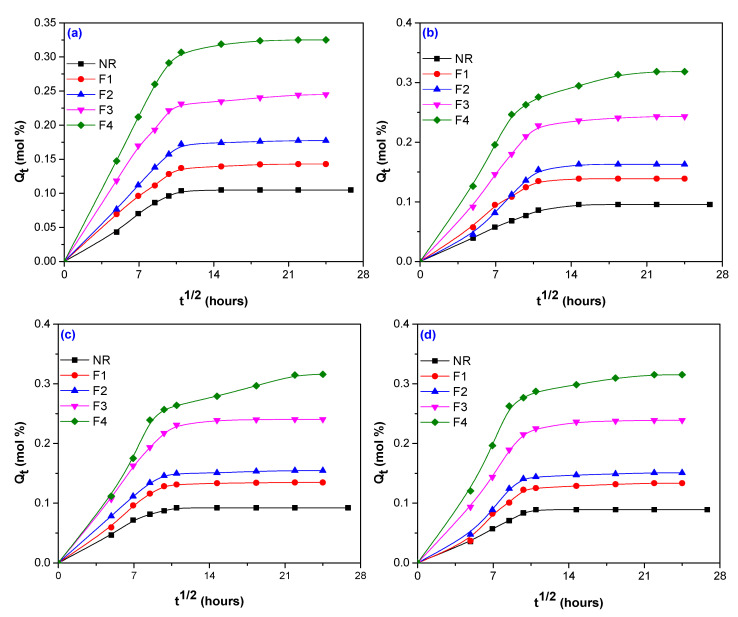
*Q_t_* variation versus immersion time for blends based on NR and flax, irradiated at 75 kGy (**a**); 150 kGy (**b**)**;** 300 kGy (**c**)**;** and 600 kGy (**d**).

**Figure 3:**
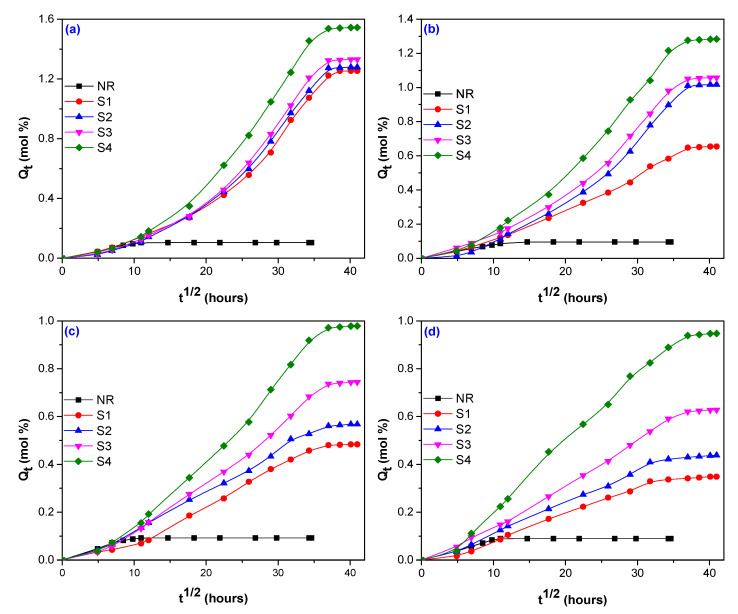
*Q_t_* variation versus immersion time for blends based on NR and sawdust, irradiated at 75 kGy (**a**); 150 kGy (**b**)**;** 300 kGy (**c**)**;** and 600 kGy (**d**).

The percent of water absorption in composites obtained by electron beam (EB) irradiation depends on three factors: the nature of the fiber (here hemp, flax and sawdust), the amount used in blends and irradiation dose. A comparison between *Q_t_* of composites with and without fibers (hemp, flax, and sawdust) and vulcanized by EB irradiation at 75, 150, 300, and 600 kGy is presented in Figure 1, Figure 2 and Figure 3.

By comparing Figure 1 and Figure 3 with Figure 2, it can be seen that the water absorption in the composites reinforced with flax (Figure 2) was faster in the first 200 h than for those with hemp (Figure 1) and sawdust (Figure 3). After that, the *Q_t_* increased progressive and reached the equilibrium after 600 h, approximately (Figure 2). Figure 1 and Figure 3 show that the *Q_t_* variation for hemp and sawdust is quite different compared to that of flax. These composites have presented a progressive *Q_t_* increasing, but not in a short immersion time as in the flax case, the equilibrium being reached after 1300 h, approximately.

All experiments have shown that the increasing of the fiber load over 5 phr, irrespective of the fiber nature, led to a significant increase of water absorption. At the irradiation doses of 75 kGy, water absorptions of 9.56%, 1.36%, and 11.93% have been obtained for composites reinforced with 5 phr hemp, flax, and sawdust, respectively. However, by increasing the irradiation dose up to 600 kGy, the water absorptions have decreased to 5.62%, 1.27%, and 3.378%, for the same loading of 5 phr hemp, flax, and sawdust. By the increasing of the fiber loading at 20 phr, *Q_t_* has increased. Thus, for irradiation doses of 75 and 600 kGy, water absorptions were obtained of 23.42% and 13.87 % for hemp, 3.65% and 3.54% for flax, 17.35% and 10.64% for sawdust. The same decreases of water absorption as in the case of 5 phr loading at the highest irradiation dose of 600 kGy, irrespective of the fiber nature, were also observed.

The hydrophilic nature of fibers is responsible for the water absorption increasing through the formation of hydrogen bonds between water and –OH groups of cellulose and hemicellulose. Thus, the increasing of the fiber load in blend leads to the increasing of hydrogen bonds number between the organic components and water molecules. Thus, water start to be accumulated in the larger gaps formed at the interface between fiber and matrix [18,54,55].

In order to attenuate or to solve the problem, chemical and physical treatments that consist of changing of the fiber surface polarity and roughness can be applied. By chemical treatments (that involves the use of alkali, acetyl, silane, benzyl, acryl, permanganate, peroxide, isocyanate, titanate, zirconate, or acrylonitrile) the fiber’s hydroxyl and carbonyl groups can be modified, new reaction centers can be created or new interacting groups that effectively interlock with the polymeric matrix at the interface can be introduced [55,56]. Physical treatments with plasma, corona, or EB lead to the formation of hydrophobic groups that increase the interfacial surface area.

In order to obtain the eco-composites that are the subject of the present paper, the EB irradiation without toxic and environmental pollutants additives was used. By EB irradiation of NR/fibers blends, the following phenomena are happening simultaneously: NR cross-linking, fibers modification and increasing of the addesion between elastomer and fibers. The composites cross-linked by EB irradiation present an increased degree of compatibility between rubber and fibers and an improved adherence to the interface between them. The number of –OH groups is reduced and as a consequence, the water absorption is restrictioned. In this way, one can correlate the reduction of water absorption degree with the irradiation dose increasing, irrespective of the fiber nature and amount used in blends [18,42,44].

In Table 4, the variation of water absorption is presented at equilibrium expressed in mol%, *Q_eq._* that corresponds to the equilibrium uptake of samples reinforced with hemp, flax, and sawdust. Based on the content of cellulose and hemicellulose (components directly involved in the water absorption process) of the three used fillers, the *Q_eq._* variation in samples should have been *Q_eq._* of flax > *Q_eq._* of hemp > *Q_eq._* of sawdust. However, data presented in Table 4, based on the experimental data, show a modified order: *Q_eq._* of hemp > *Q_eq._* of sawdust > *Q_eq._* of flax. So, the smallest values for equilibrium water uptake were obtained by the composites reinforced with flax at all irradiation doses.

**Figure 4:**
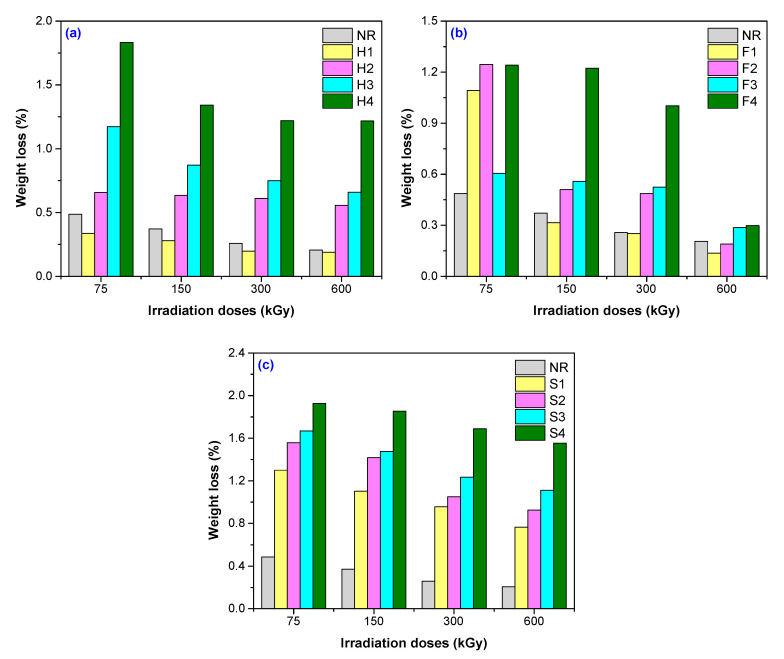
Weight loss variation for NR samples reinforced with hemp (**a**), flax (**b**)**,** and sawdust (**c**)**.**

After the equilibrium of water uptake was reached, samples were removed from water, dried until constant weight in laboratory oven and reweighed in order to establish if there were mass losses due to the exit of the un-trapped fibers in the polymer matrix. As it can be seen from Figure 4, the irradiation dose increasingly lead to the decreasing of weight loss for all three types of composites. So, we can assume that the irradiation dose increasingly favors the adhesion between the rubber and natural fibers.

In Figure 4, in which it is shown that the weight loss expressed in % after reaching the equilibrium can be observed as the same tendency to having the lowest values for flax based composites. The decreasing weight loss order is, in this case: weight loss of sawdust > weight loss of hemp > weight loss of flax. By comparing the results obtained for reinforced composites with the sample of NR without any filler, it can be observed that the smallest weight loss was for NR/flax composites also. We can conclude that the adhesion between elastomer and fiber was more favorable in the case of flax compared to the other two types of fillers. So, the water absorption depends on both fiber type (with different content in cellulose and hemicellulose) and irradiation dose. Discussion can be done with respect of cellulose because is the main common element of the three types of fibers considered. There are several studies that present the effects of electron beam irradiation on cellulose [31,44,57]. Over 20 different cellulose macro-radicals, including primary and secondary species, can be formed and distinguished. Some of them are thermodynamically favored and, thus, are far more likely to be created [44,58,59,60]. By EB irradiation of fibers, radicals having localized unpaired electrons from cellulose are formed, especially in the positions 1, 2, 3, 4, and 5 of the pyranose ring. They are originated from hydrogen abstraction from the mentioned positions and from the OH group [44,61]. Radicals formed in the positions 2 and 3 may form other radicals by loss of a water molecule. Other radical species can be formed by chain scission, by cleavage of a glycosidic bond or from β-fragmentation of an oxygen-centered radical resulting from cleavage of a glycosidic bond [44,61]. In cellulose molecules, free radicals are formed by C–H, C–O, and C–C bond cleavages by hydrogen abstraction, chain scission, and cycle opening. The radical species that are formed during the EB irradiation are involved in grafting reaction of natural filler on the NR matrix and lead to the increasing of adhesion between them [62,63]. In this way, it can be explained that the values obtained for water absorption in our experiments depend both on the fiber type (with different content in cellulose and hemicellulose) and irradiation dose.

### 3.2. Mechanism of Water Transport

There are three important factors considered as being of interest when are investigated the transport mechanisms of liquids, especially water, in composites based on rubber and fibers: the chemical nature of polymeric matrix and fiber and the compatibility and interfacial adhesion between them [64]. In the particular case of composites based on NR and natural fibers, the latter are mostly responsible for water absorption because the rubber matrix is hydrophobic, having a reduced absorbing potential (the water uptake at time t, *Q_t_* < 0.1%) due to the weak presence of proteins. The water absorption in polymers is conditioned by the availability of free nanosized holes as well as the polar sites. The penetration of water into fibers occurs through the micropores present on their surface. The waxy materials present on the fiber additionally help to reduce the water retention.

The absorbed water in the rubber composites reinforced with fibers will be found in two different stages: unbonded in the nano-holes of the rubber and bonded to the rubber and fibers through hydrogen bonds [18].

In the present study, the mechanism of water transport was analyzed using the following Equation (2) [64]:

(2) $$\log\frac{Q_{t}}{Q_{eq.}}=\log k+n\log t$$

where, *Q_t_* and *Q_eq._* are the mol% water uptake at time *t* and at equilibrium, respectively, *k* and *n* are diffusion parameters that depend on the nature of the materials and interfacial adhesion.

The values of diffusion parameters, *k* and *n*, have been found from the slope and y-intercept of the log *Q_t_*/*Q_α_* vs. log*t* plots. The values of *n* determine the type of the water transport mechanism that follow the kinetics and mechanisms described by Fick’s theory [21]. Moisture diffusion in polymeric composites has been shown to be Fickian as well as non-Fickian in character, as follows: (a) Fickian diffusion (Case I), corresponding to a slow water mobility compared with the one of the polymer chain segments, case in which there is no interaction and the equilibrium state within the polymer is rapidly attained. The *n* value is 0.5; (b) Anomalous diffusion, corresponding to a comparable water mobility with the loosening of the polymer chain segments. The *n* value is between 0.5 and 1; (c) Non-Fickian diffusion (Case II where n = 1 and super Case II with n > 1), corresponding to a faster water mobility than all other relaxations [64,65]. This can happen due to composites complex mechanisms that depend on many factors such as: volume fraction of fibers, void volume, additives, humidity, temperature, orientation of reinforcement, nature of fiber (that is permeable or impermeable), area of exposed surfaces, diffusivity, reaction between water and matrix, and finally surface protection [18].

**Figure 5:**
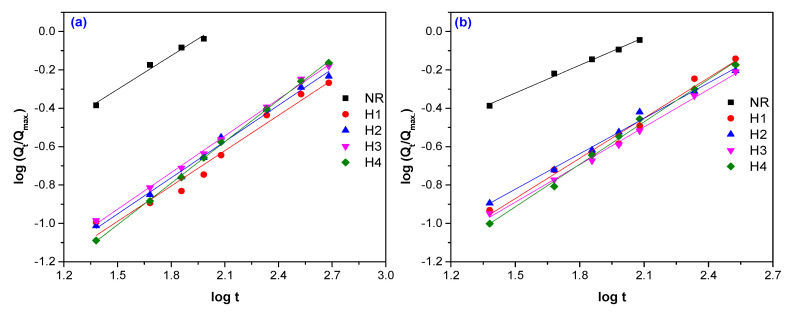

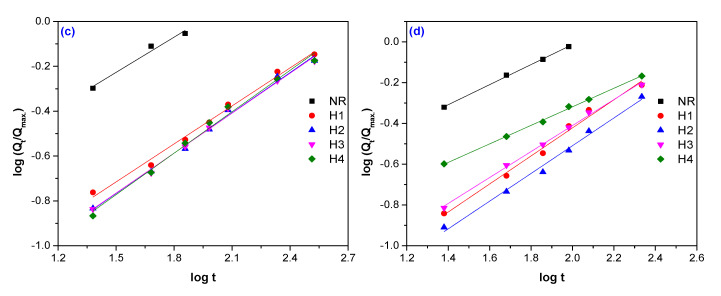
Determination of the diffusion parameters *n* and *k* for NR samples reinforced with hemp and vulcanized by electron beam (EB) irradiation at 75 kGy (**a**), 150 kGy (**b**), 300 kGy (**c**)**,** and 600 kGy (**d**).

**Figure 6:**
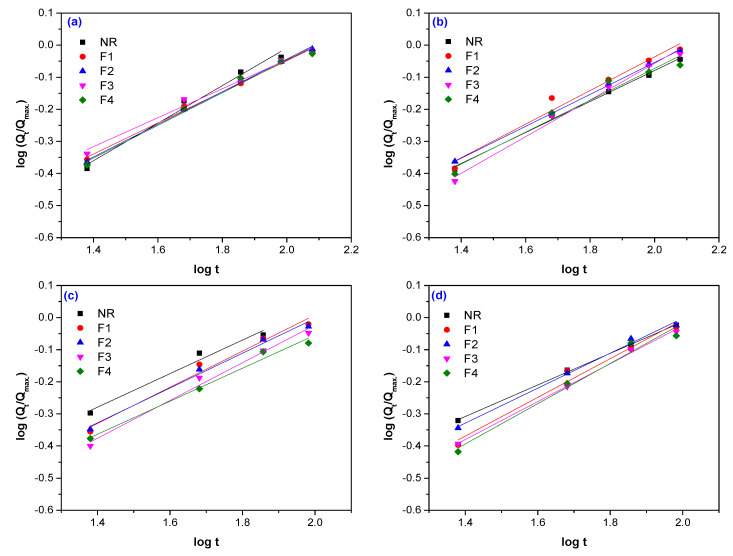
Determination of the diffusion parameters *n* and *k* for NR samples reinforced with flax and vulcanized by EB irradiation at 75 kGy (**a**), 150 kGy (**b**), 300 kGy (**c**)**,** and 600 kGy (**d**).

**Figure 7:**
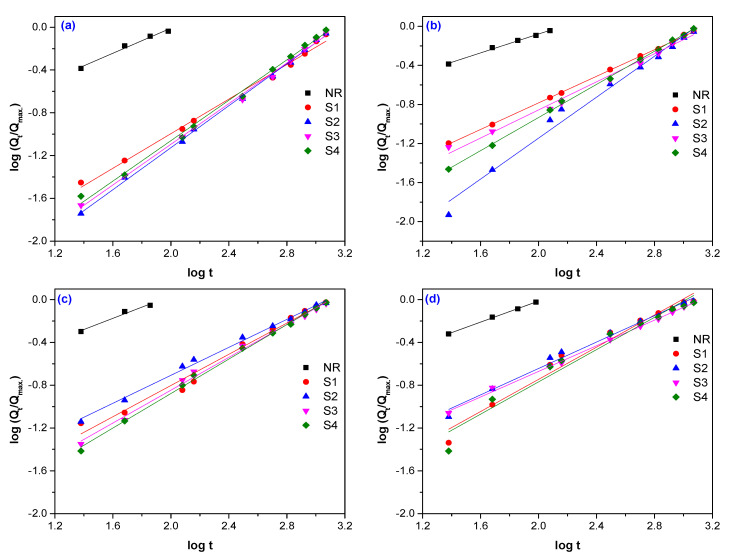
Determination of the diffusion parameters *n* and *k* for NR samples reinforced with sawdust and vulcanized by EB irradiation at 75 kGy (**a**), 150 kGy (**b**), 300 kGy (**c**)**,** and 600 kGy (**d**).

Typical plots of experimental data fitted by Equation (2), are presented in Figure 5, Figure 6 and Figure 7. The values of difussion parameters *n* and *k* have been evaluated by linear regression analysis applied on plots presented in Figure 5, Figure 6 and Figure 7 and the results are listed in Table 5.

In Table 5, it can be seen that for unreinforced NR the smallest value of *n* (*n* = 0.483) has been obtained at the irradiation dose of 150 kGy and the biggest one (*n* = 0.586) at 75 kGy. It seems that the increasing of irradiation dose brings the water absorption behavior closer to the Fikian one. Thus, the water molecule’s mobility is lower than that of rubber chain’s segments and the equilibrium is attended faster, as is also shown in Figure 1, Figure 2 and Figure 3. The values of *n* corresponding to the composites are different, depending on the fiber nature used for reinforcement and, as a consequence, on the fibers chemical composition. As it was previously mentioned, the components mainly responsible for water absorption of natural fibers are cellulose and hemicellulose. The higher their percentage in the material, the higher the amount of water absorbed. Even if the flax is rich in cellulose and hemicellulose, its behavior at radiation field is over the hemp or sawdust one. The *n* value doesn’t vary too much with the flax load, irrespective of the radiation dose and presents values between 0.451 (NR/15 phr, 75 kGy) and 0.627 (NR/20 phr, 600 kGy). These values are closer to Fickian diffusion than to Anomalous. In the case of hemp use, it can be observed an increasing of *n* values with both fiber load and irradiation dose, the behavior being Anomalous. Only for the amount of 20 phr were obtained values of 0.722 at 75 kGy and 0.454 at 600 kGy, respectively. In the case of sawdust use, the behavior is near the non-Fickian one: *n* = 0.809 (5 phr) and *n* = 0.945 (20 phr), both at 75 kGy. With the irradiation dose increasing, water diffusion becomes Anomalous. Deviations from Fickian diffusion can be attributed to moisture diffusion, desorption, osmotic cracking, or micro-cracks formation [64]. In our experiments, the *n* values of all reinforced composites, that are composed of two phases—cross-linked elastomer and bound or unbound fibers, are higher than of NR unreinforced, that is composed of only one phase—cross-linked elastomer. The water absorption in reinforced composites is happening in polymer matrix (NR, in our case), filler (hemp, flax, and wood sawdust) but also at the elastomer–fiber interface. Hemp, flax, and sawdust used in the experiments are fibers having strong hydrophilic character. The cross-linked composites reinforced with these types of fibers absorb higher amounts of water through the hydrogen bonds created between water and the –OH groups in cellulose and hemicellulose. Absorption difference between the composites reinforced with the three types of fibers, reflected through the n values, is due the adhesion degree between elastomer and fiber that depends on fiber nature and loading and also on irradiation dose. The stronger the interaction between the fiber and the elastomer, the smaller the amount of water absorbed and also the lower the value of the n parameter.

The diffusion parameter *k* is a constant of material that varies as a function of structure and offer information about its interaction with water. Low values of *k* show a weak interaction between composite and water that means poor water absorption. *k* values in Table 5 are under 0.1 for all kinds of samples, except the ones reinforced with flax. Although, according to these results, there is a higher interaction between NR and NR/flax with water and the absorption is lower in these samples (Table 4) due to the elastomer structure (in NR samples) and the better interaction between elastomer and flax, compared to hemp and sawdust. Water cannot easily penetrate the NR and the NR/flax structures.

### 3.3. Transport Coefficients

Water diffusion experiments are useful to study the rubber/fiber interaction. Water molecule is small compared with the rubber based composites macromolecule and its transport is realized through a diffusion process that takes place in two stages. At first, the water molecules penetrate and are sorbed in the rubber/filler matrix, after that being diffused in it (intermingling of the two systems). Consequently, the overall water transport is determined by the difference in amount of the penetrant molecules between the two phases. The water absorption tends to the equilibrium, the stage that is strongly connected to the material nature and is expressed through the following coefficients: diffusion coefficient (*D*), the intrinsic diffusion coefficient (D*), the sorption coefficient (*S*), and the permeation coefficient (*P*) [64].

In Figure 1, Figure 2 and Figure 3 (the sorption curves *Q_t_* vs. t^1/2^ for composites reinforced with hemp, flax and sawdust), the diffusion coefficient *D* was calculated from the slope *θ* of the initial linear portion, using the following Equation (3) [66,67,68]:

(3) $$D=\pi {\left(\frac{h\theta }{4Q_{eq.}}\right)}^{2}$$

where *h* is the thickness of the polymeric composite and *Q_eq_*_._ is the equilibrium water uptake, mol%.

During absorption experiments, due to the fiber presence in blends, a significant swelling of composites was observed. Thus, a correction of the diffusion coefficients under swollen conditions was required and was made by calculating the intrinsic diffusion coefficient D* from the volume fraction of polymer in the swollen composite, through the following Equation (4) [66,69]:

(4) $$D^{*}=\frac{D}{\Phi^{7/3}}$$

where D is the diffusion coefficient calculated as above and Φ is the volume fraction of solvent (water), calculated as the following Equation (5) [66,70]:

(5) $$\Phi =\frac{W_{1}/\rho_{1}}{W_{1}/\rho_{1}+W_{2}/\rho_{2}}$$

where *W*_1_ and *ρ_1_* are the weight and density of the polymer sample respectively and *W_2_* and *ρ_2_* are the weight and density of the solvent (water), respectively.

Densities of composites were determined by hydrostatic weighing method, according to SR ISO 2781/2010. By this method, the volume of a solid sample is determined by comparing the weight of the sample in air with the weight of the sample immersed in a liquid of a known density (ethyl alcohol). The volume of the sample is equal to the difference between the two weights divided by the density of the liquid. The obtained values of densities for all types of composites were between 0.95 and 1.01 g/cm^3^ and for the NR itself of 0.94 g/cm^3^.

Permeation is a collective process of diffusion and sorption. Permeation coefficient, *P*, is defined as being, through the following Equation (6) [66,68]:

(6) $$P=D^{*}\times S$$

where, D* is the intrinsic diffusion coefficient and S the sorption coefficient.

The permeation of water into the composite samples depends on the diffusivity as well as on the sorption. The sorption coefficient, *S*, is calculated using the following Equation (7) [66,71]:

(7) $$S=\frac{M_{eq.}}{M_{0}}$$

where, *M_eq._* is the mass of the solvent at equilibrium swelling and *M_0_* is the initial polymer mass.

The calculated values of diffusion coefficient (*D*), volume fraction of water (Φ) and intrinsic diffusion coefficient (D*) for all types of composites reinforced with hemp, flax, and sawdust are presented in Table 6.

The diffusion coefficient, *D*, is the most important parameter of Fick model that describe the water molecules capacity to penetrate the polymer matrix. Also, the diffusion coefficient offer information about the water absorption velocity and, its values allow to quantitatively evaluation of the water absorption process [72,73].

As seen in Table 6 the values of *D*, Φ, and D* present visible variations as a function of filler nature (hemp, flax, and sawdust) and loading in blends but also with the irradiation dose. The parameter D decreases in the order of flax > hemp > sawdust, which also represents the order in which it decreases the percentage of cellulose and hemicellulose that are directly responsible for the fiber hydrophilic character. The increasing of fiber loading in the composite leads to the increasing of *D* values. This result can be attributed to an increased heterogeneity in sample as the fiber loading increased. As other authors have mentioned [72,73], the diffusion characteristics in a diffuse polymeric system may suffer significant changes in time. Two mechanisms were offered as being possible: (1) the polymer structure suffers a relaxation during water diffusion; (2) during water diffusion, the swelling is connected with internal stresses occurrence in some regions that affect other neighboring regions in the composite. It was concluded that both relaxation and internal stresses have the same effect on the diffusion coefficient and, consequently, on the mechanical properties of the composites [72,73].

**Figure 8:**
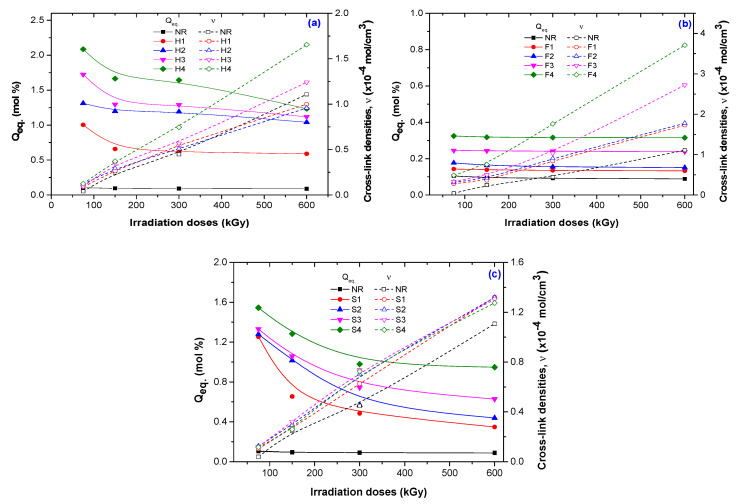
*Q_eq._* (mol%) vs. cross-link densities (ν) [42,44,45] of NR samples reinforced with hemp (**a**), flax (**b**), and sawdust (**c**).

As can be seen from Figure 8, the increasing of cross-link density with the irradiation dose [42,44,45] lead to the decreasing of Q_eq_ especially for samples reinforced with hemp (Figure 8a) and sawdust (Figure 8c).

As it was already mentioned, the diffusion coefficient *D* must be corrected through the intrinsic diffusion coefficient D*. In order to do that, the volume fraction Φ in the swollen composite was also determined. The value of the volume fraction is a measure of the cross-linking density: as the values of the volume fraction decrease, the cross-linking density also decreases [66]. The highest values of the volume fraction were obtained for the composites reinforced with flax and the lowest for those reinforced with sawdust. Also, the increasing of irradiation dose has lead to the cross-linking degree increasing [66] and, as a consequence, to the volume fraction increasing.

The registered results regarding the variation of diffusion coefficients, D and D*, can be explained in the same way as in the case of diffusion parameters *n* and *k* variation: as the fiber volume fraction increases, the maximum water absorption also increases. Samples with high fiber content swell much more than the polymer matrix, micro-cracks at the interface between fiber and matrix are formed and the diffusion of water is facilitated. The water transport can be explained by the activation of the capillarity mechanism: the water molecules flow through the fiber–matrix interface leading to a great diffusivity [74,75,76].

The permeation coefficient, *P*, is equal with the steady rate of permeation of water molecules through a layer of limitted thikness and its values depends on haw fast the water molecule moves through the polymer mass (diffusion coefficient, *D*) and on the mass of water absorbed per polymer mass at the saturation point (sorption coefficient, *S*) [73]. Increased values of *P* and *S* are usually connected with high absorption capacities.

For the evaluation of the collective process of diffusion and sorption in the composites reinforced with natural fibers (hemp, flax, and sawdust), *P* and *S* coefficients were calculated. The results are presented in Table 7.

As seen in Table 7, the permeation coefficient values significant increase with the fiber loading and decrease with the irradiation dose. In the case of this coefficient, the hydrophilic behavior of the fibers generates a different order than in the case of other coefficients and parameters studied before, as follows: *P* of hemp > *P* of flax > *P* of sawdust.

The irradiation dose increasing lead to the cross-linking degree increasing which, in turn, leads to the better interaction between the fiber and the matrix of NR due to the –OH groups reducing in the fibers. The increasing of cross-linking degree with the irradiation dose was already demonstrated in previous works [41,42,43,44,45] in which were characterized composites reinforced with hemp, flax, and sawdust in order to establish this connection. Thus, the cross-linking degrees of NR samples containing 5 phr of flax, hemp, and sawdust and irradiated at 75 kGy were of 0.2792 × 10^−4^ mol/cm^3^, 0.0898 × 10^−4^ mol/cm^3^, and 0.1033 × 10^−4^ mol/cm^3^, respectively. For the highest irradiation dose of 600 kGy the cross-linking degrees of similar samples were of 1.7298 × 10^−4^ mol/cm^3^, 0.9990 × 10^−4^ mol/cm^3^, and 1.3208 × 10^−4^ mol/cm^3^, respectively.

Also in Table 7, it can be seen that the sorption coefficient values, irrespective of the irradiation dose, are under the permeation coefficient values. This result shows that the dominant process is diffusion and not sorption.

The dynamic of these coefficients is also connected with the same causes as in the case of diffusion parameters and coefficients evaluated previously: hydrophilic character of fibers in contrast with the hydrophobic character of NR matrix correlated with the fiber loading that generate differences of homogeneities in the same sample and different absorption mechanisms of water due to the appearance of non-uniform swelling deformation and stress areas.

## 4. Conclusions

Natural rubber composites reinforced with hemp, flax, and wood sawdust in amounts between 5 and 20 phr were obtained by electron beam irradiation, at room temperature, with doses between 75 and 600 kGy. The kinetics of water absorption in these materials were studied and the water diffusion was being analyzed through Fick’s law. The obtained results showed that all parameters and coefficients connected with the water absorption through diffusion and sorption mechanisms depend on the fiber nature, amount used in blends, and irradiation dose.

Dynamics of water absorption, in the case of flax, was found as being different than the cases of hemp and sawdust. NR/flax composites presented a fast absorption in the first 200 h and reached the equilibrium after 600 h. NR/hemp and NR/sawdust presented a progressive absorption and the equilibrium was reached after 1300 h. Increasing the fibers load, increases the water absorption, while increasing the irradiation dose, decreases the water absorption. The smallest weight loss was also in the case of NR/flax composites. Based on these results it can be concluded about a better adhesion between elastomer and flax, compared to the other two types of fillers.

Based on the values of diffusion parameter *n*, the type of water transport mechanism was established. Thus, in the case of NR/flax composites irrespective of the flax load and radiation dose the diffusion was closer to Fickian than to Anomalous. The NR/hemp composites showed mostly Anomalous behavior and NR/sawdust was a mix between non-Fickian at low irradiation dose and Anomalous. Deviations from Fickian diffusion can be attributed to moisture diffusion, desorption, osmotic cracking, or micro-cracks formation. The interaction between the composite and water was evaluated through diffusion parameter *k* that was under 0.1 for all kind of samples, except the ones reinforced with flax. According to all these results, the interaction between elastomer and flax is better than between elastomer and hemp or sawdust and the water absorption is low. Water cannot easily penetrate the NR and the NR/flax structures.

## Figures and Tables

**Table 1:** The properties of materials used in experiments.

Material	Function	Properties
natural rubber of Crep 1X type	matrix	Mooney viscosity is 74 ML_1+4_ at 100 °C, 0.32 wt.% volatile materials, 0.38 wt.% nitrogen, 0.22 wt.% ash, 0.021 wt.% impurities
irganox 1010	antioxidant	melting point of 40 °C, 1.15 g/mL density (at 40 °C), 98% active ingredient
polyethylene glycol PEG 4000	lubricant and plasticizer	1.128 g/cm^3^ density, 4–8 °C melting point range
hemp fiber	filler	thread length of max 3 mm; composition: 70.2–74.4 wt.% cellulose, 17.9–22.4 wt.% hemicellulose, 3.7–5.7 wt.% lignin, 0.8 wt.% wax, 10.8 wt.% water, 7.0 wt.% others [35,36,37].
flax wastes	filler	thread length of max 3 mm; composition: 71–75 wt.% cellulose, 18.6–20.6 wt.% hemicellulose, 2.2 wt.% lignin, 1.7 wt.% wax, 10.0 wt.% water, 6.0 wt.% others [35,36,37].
wood sawdust	filler	ground of max mesh 250–270; composition: 38–51 wt.% cellulose, 17–38 wt.% hemicellulose, 21–31 wt.% lignin, 1.5 wt.% wax, 6.7 wt.% water, 3.0 wt.% others [38,39,40].

**Table 2:** Blends composition, ingredients and loading expressed in Parts per Hundred Rubber (phr).

Ingredients	Loading (phr)
Natural rubber	100
PEG 4000	3
Irganox 1010	1
Filler (hemp, flax and wood sawdust)	0–20

**Table 3:** Sample codes.

Natural Rubber without Filler Addition			NR
Filler Amount (phr)	Hemp	Flax	Sawdust
5	H1	F1	S1
10	H2	F2	S2
15	H3	F3	S3
20	H4	F4	S4

**Table 4:** *Q_eq._* (mol%) variation for NR, NR/hemp, NR/flax, and NR/sawdust samples

	Filler Amount (phr)	Irradiation Doses			
		75 kGy	150 kGy	300 kGy	600 kGy
NR	no	0.105	0.095	0.092	0.089
NR/ Hemp	5	1.004	1.313	1.725	2.085
	10	0.658	1.197	1.297	1.666
	15	0.615	1.192	1.292	1.644
	20	0.590	1.041	1.120	1.235
NR/ Flax	5	0.143	0.177	0.245	0.325
	10	0.139	0.163	0.243	0.318
	15	0.135	0.155	0.241	0.316
	20	0.133	0.151	0.239	0.315
NR/ Sawdust	5	1.254	1.279	1.331	1.545
	10	0.654	1.017	1.058	1.283
	15	0.484	0.568	0.744	0.979
	20	0.348	0.438	0.628	0.947

**Table 5:** Values of *n* and *k* × 10^2^ parameters as a function of irradiation dose.

	Filler (phr)	Irradiation Doses							
		75 kGy		150 kGy		300 kGy		600 kGy	
		*n*	*k* × 10^2^	*n*	*k* × 10^2^	*n*	*k* × 10^2^	*n*	*k* × 10^2^
NR	no	0.586	6.60	0.483	9.03	0.523	9.73	0.492	10.06
NR/ Hemp	5	0.614	1.23	0.699	1.21	0.565	2.74	0.688	1.59
	10	0.630	1.27	0.616	1.80	0.599	2.16	0.680	1.35
	15	0.638	1.31	0.652	1.36	0.593	2.21	0.637	2.07
	20	0.722	0.81	0.738	0.95	0.616	2.01	0.454	5.92
NR/ Flax	5	0.485	9.58	0.524	8.24	0.563	7.61	0.604	6.10
	10	0.508	8.70	0.497	8.94	0.542	8.18	0.545	8.10
	15	0.451	11.26	0.571	6.33	0.589	6.31	0.591	6.20
	20	0.509	8.63	0.496	8.61	0.515	8.24	0.627	5.35
NR/ Sawdust	5	0.809	0.24	0.681	0.72	0.730	0.55	0.752	0.56
	10	0.982	0.08	1.048	0.06	0.654	0.96	0.618	1.32
	15	0.951	0.10	0.720	0.51	0.768	0.41	0.599	1.35
	20	0.945	0.11	0.849	0.23	0.806	0.32	0.755	0.53

**Table 6:** Values of *D* × 10^9^ m^2^ s^−1^, Φ and D* × 10^9^ m^2^ s^−1^ as a function of irradiation dose.

	Filler (phr)	Irradiation Doses											
		75 kGy			150 kGy			300 kGy			600 kGy		
		*D* × 10^9^	Φ	D* × 10^9^	*D* × 10^9^	Φ	D* × 10^9^	*D* × 10^9^	Φ	D* × 10^9^	*D* × 10^9^	Φ	D* × 10^9^
NR	no	9.41	0.983	9.80	6.03	0.984	6.27	5.51	0.985	5.72	7.99	0.983	8.32
NR/ Hemp	5	2.34	0.857	3.36	2.32	0.902	2.95	2.41	0.907	2.81	2.08	0.911	2.58
	10	2.47	0.820	3.92	2.43	0.833	3.73	2.36	0.834	3.61	2.11	0.851	3.08
	15	2.48	0.772	4.32	2.44	0.818	3.90	2.37	0.819	3.78	2.36	0.839	3.56
	20	2.98	0.733	6.16	2.56	0.775	4.64	2.44	0.777	4.39	2.37	0.823	3.70
NR/ Flax	5	10.24	0.976	10.84	9.93	0.977	10.51	9.86	0.977	10.40	9.91	0.977	10.46
	10	10.71	0.970	11.49	9.76	0.965	10.62	9.48	0.974	10.09	9.63	0.974	10.23
	15	9.05	0.958	10.01	8.26	0.958	9.12	7.62	0.959	8.39	7.06	0.959	7.79
	20	10.58	0.945	12.08	8.21	0.946	9.35	7.26	0.946	8.27	6.38	0.946	7.26
NR/ Sawdust	5	0.427	0.830	0.661	0.402	0.903	0.510	0.201	0.927	0.240	1.20	0.946	1.37
	10	0.445	0.823	0.701	0.417	0.854	0.602	0.653	0.913	0.807	1.12	0.932	1.33
	15	0.483	0.816	0.777	0.441	0.848	0.647	0.642	0.888	0.848	0.719	0.904	0.911
	20	0.509	0.789	0.884	0.493	0.818	0.787	0.627	0.855	0.903	0.801	0.859	1.14

**Table 7:** Values of *P* × 10^10^·m^2^ s^−1^ and *S* (%) as a function of irradiation dose.

	Filler (phr)	Irradiation Doses							
		75 kGy		150 kGy		300 kGy		600 kGy	
		*P* × 10^10^	*S*	*P* × 10^10^	*S*	*P* × 10^10^	*S*	*P* × 10^10^	*S*
NR	no	1.85	0.019	1.08	0.017	0.95	0.017	1.53	0.018
NR/ Hemp	5	6.07	0.181	3.49	0.119	3.12	0.111	2.74	0.106
	10	9.28	0.237	8.05	0.216	7.75	0.215	5.70	0.188
	15	14.08	0.311	9.11	0.234	8.81	0.233	7.18	0.202
	20	23.15	0.376	13.92	0.301	13.03	0.296	8.29	0.222
NR/ Flax	5	2.79	0.026	2.62	0.025	2.53	0.024	2.53	0.024
	10	3.67	0.032	3.93	0.038	2.84	0.028	2.78	0.027
	15	4.41	0.044	3.99	0.044	3.64	0.043	3.35	0.043
	20	7.08	0.059	5.36	0.057	4.71	0.057	4.12	0.057
NR/ Sawdust	5	1.49	0.226	0.61	0.118	0.21	0.087	0.86	0.063
	10	1.62	0.230	1.11	0.183	0.83	0.102	1.04	0.079
	15	1.86	0.240	1.23	0.191	1.14	0.134	1.03	0.113
	20	2.46	0.278	1.82	0.231	1.59	0.176	1.95	0.171
